# Hospital mortality in older patients in the Brazilian Unified Health System, Southeast region

**DOI:** 10.11606/S1518-8787.2018052000146

**Published:** 2018-07-17

**Authors:** Paula Cordeiro, Mônica Martins

**Affiliations:** I Fundação Oswaldo Cruz. Escola Nacional de Saúde Pública Sérgio Arouca. Programa de Pós-Graduação em Saúde Pública. Rio de Janeiro, RJ, Brasil; II Fundação Oswaldo Cruz. Escola Nacional de Saúde Pública Sérgio Arouca. Departamento de Administração e Planejamento em Saúde. Rio de Janeiro, RJ, Brasil

**Keywords:** Aged, Cardiovascular Diseases, Hospital Mortality, Risk Factors, Quality of Health Care, Unified Health System, Idoso, Doenças Cardiovasculares, Mortalidade Hospitalar, Fatores de Risco, Qualidade da Assistência à Saúde, Sistema Único de Saúde

## Abstract

**OBJECTIVE:**

To evaluate factors associated with hospital death in older inpatients for specific diseases of the circulatory system in the Brazilian Unified Health System considering the risk-adjusted hospital mortality as an indicator of effectiveness.

**METHODS:**

The data were extracted from the Brazilian Hospital Information System. A total of 385,784 hospitalizations of older were selected for hypertensive diseases, ischemic heart disease, congestive heart failure, and stroke in the Brazilian Southeast region between 2011 and 2012. Age, sex, emergency admission, principal diagnosis, and two comorbidity indexes were included in the logistic regression for the risk adjustment of hospital death. The analyses were developed at two levels: hospitalization and hospital.

**RESULTS:**

A greater chance of death was observed with increasing age, emergency hospitalizations, stroke, presence of comorbidities, especially pneumonia and weight loss, hospitalizations for clinical care, and use of intensive care units. The risk-adjusted hospital mortality rate was 11.1% in for-profit private hospitals, 12.3% in non-profit private hospitals, and 14.4% in public hospitals, but there was great variability among the hospitals. The hospital standardized mortality ratio (ratio between observed and predicted deaths) ranged from 103.3% in non-profit private hospitals to 118.2% in for-profit private hospitals.

**CONCLUSIONS:**

Although the information source has its shortcomings, the ability for discrimination of the risk adjustment model was reasonable. The variability in the risk-adjusted hospital mortality was great and comparatively higher in for-profit private hospitals. Despite the limits, the results favor the use of the risk-adjusted hospital mortality in the monitoring of the quality of hospital care provided to the older adult.

## RESUMO

**OBJETIVO:**

Avaliar fatores associados ao óbito hospitalar em idosos internados por doenças do aparelho circulatório específicas no Sistema Único de Saúde, considerando a mortalidade hospitalar ajustada como indicador de efetividade.

**MÉTODOS:**

As informações foram extraídas do Sistema de Informações Hospitalares. Foram selecionadas 385.784 internações de idosos por doenças hipertensivas, doença isquêmica do coração, insuficiência cardíaca congestiva e doenças cerebrovasculares, na região Sudeste entre 2011 e 2012. Idade, sexo, admissão de emergência, diagnóstico principal e dois índices de comorbidade foram incluídos na regressão logística para o ajuste do risco de óbito. As análises foram desenvolvidas em dois níveis: internação e hospital.

**RESULTADOS:**

Observou-se maior chance de morrer nas idades mais avançadas, nas internações de urgência, por doenças cerebrovasculares, com registro de comorbidade, especialmente pneumonia e perda de peso, nas internações para cuidado clínico e com uso de unidades de terapia intensiva. A taxa de mortalidade hospitalar ajustada foi 11,1% nos hospitais privados, 12,3% nos filantrópicos e 14,4% nos públicos, mas houve grande variabilidade entre hospitais. A razão de mortalidade hospitalar ajustada (razão entre óbitos observados e preditos) variou entre 103,3% nos hospitais filantrópicos e 118,2% nos hospitais privados.

**CONCLUSÕES:**

Embora haja insuficiências na fonte de informação, a capacidade de discriminação do modelo de ajuste de risco mostrou-se razoável. A variabilidade na mortalidade hospitalar ajustada foi ampla e comparativamente maior nos hospitais privados. Apesar dos limites, os resultados favorecem o uso da mortalidade hospitalar ajustada por risco no monitoramento da qualidade do cuidado hospitalar prestado ao idoso.

## INTRODUCTION

In most countries, the growth of the older population is one of the most significant social transformations of the twenty-first century, with impacts for society in several contexts, from the labor market and social security to the growing demand for social care, health care, and health services^1^ ^,^ ^2^. Regarding the Brazilian health system, the health care model for older adults faces several challenges, especially difficulties in coordinating and integrating care, the lack of qualified professionals, the unmet demand for specialized outpatient services, and the insufficient supply of home care^3^ ^,^ ^4^. In addition, the model focused on the main complaint associates all signs and symptoms to only one diagnosis, compromising the effectiveness of the care provided to these patients, which can result in avoidable hospitalizations or worsening of the condition^3^ ^,^ ^4^.

In this scenario, marked by the increase in life expectancy and the prevalence of chronic diseases^1^, the agenda for quality improvement of health services has notably incorporated these two issues into the list of problems and challenges. Consequently, the need for methodological approaches is recognized as crucial to scale the effects of the multiplicity of chronic diseases that more intensely, but not exclusively, affect older adults^5^ ^,^ ^6^.

The high rates of hospitalization and the trend of more frequent and prolonged hospitalizations highlight the importance of the monitoring in order to improve the quality of the hospital care provided to the older population^4^. From this perspective, the analysis of clinical performance indicators provides initial elements for improvement actions. Among these indicators, hospital death is an indirect measure of the outcome, which indicates problems in the quality of care and which is widely used in several countries^7^. However, the validity of this type of indicator is conditioned, among other aspects, to risk adjustment for the control of confounding factors related to the greater severity of the case and the worse prognosis^6^ ^,^ ^7^.

In particular, the adequate risk adjustment of clinical performance indicators constructed for the older population has gained importance given the overlapping morbidity, physiological weaknesses, longer length of stay, and the intense use of health resources and services^5^. Although its relevance is recognized, few methods of risk adjustment have been conceived and tested in this age group^5^ ^,^ ^6^ ^,^ ^8^. In addition, other methodological issues emerge, such as the need to use specific and epidemiologically relevant indicators^9^.

In Brazil, for more than 10 years, studies have emphasized the importance of hospital mortality to assess and monitor the quality of hospital care provided to persons aged 60 years or more^9^ ^,^ ^10^ ^,^ ^11^. However, despite the relevance of the subject, there are few studies in the country. Information on hospital morbidity indicates diseases of the circulatory system as the main cause of hospitalization of older adults^a^. In this perspective, the objective of this study was to evaluate factors associated with hospital death in older inpatients for specific diseases of the circulatory system in the Brazilian Unified Health System (SUS) in the Southeast region, exploring the risk-adjusted hospital mortality as an indicator of the quality, a dimension of the effectiveness of the hospital care provided.

## METHODS

This is a cross-sectional, observational study based on secondary data. Hospital death, as the measure of the outcome care, was the main issue; it was also used as an indicator of clinical performance – risk-adjusted hospital mortality rate (number of predicted deaths divided by hospitalizations per 100), which expresses the dimension of the effectiveness of the care provided. In the study design, length of stay (measure of technical efficiency) was considered a variable that explained hospital mortality, since it can express greater severity, occurrence of avoidable complications, and variations in practices among hospitals or provision of post-hospital care^12^.

The source of the data used was the Hospital Information System (SIH) of the SUS. This system is the main secondary database on hospital production of national scope. Although it is linked to the transfer of financial resources and the shortcomings reported in the literature, the SIH is used to evaluate clinical performance and to describe the morbidity profile of the hospital^13^. The SIH has information on the demographic characteristics of the patients (age, sex), principal and secondary diagnoses, medical specialty, type of admission, days of stay, use of intensive care unit (ICU), type of outcome, and amount paid for the procedure performed^14^. The data necessary for the development of this study have been stored in reduced files, accessible in the DATASUS portal (http://www2.datasus.gov.br/DATASUS/). From these files, we extracted only the records called type 1 “Hospitalization Authorization” (AIH), defined as normal, which would correspond to hospitalizations whose length of stay is less than 45 days^14^. However, hospitalizations with stay over 45 days were present among the type 1 AIH included (2,835 cases – 0.7%). The data recorded on the type 5 AIH, which corresponds to long-term care, were excluded to avoid duplication of records, especially in older adults given the greater probability of sequential hospitalizations or readmissions.

The initial sample of the universe included hospitalizations of older adults (≥ 60 years) in the SUS, in the Southeast region, between 2011 and 2012 (Figure). Considering the hospital morbidity profile of the older population and the number of hospitalizations and deaths, we selected the hospitalizations whose principal diagnosis corresponded to Chapter IX of the Circulatory System Diseases of the 10th revision of the International Statistical Classification of Diseases and Related Health Problems (ICD-10). We sequentially applied the same criterion (number of hospitalizations) to the hospitalizations in chapter IX; therefore, the study universe was focused on the following specific reasons for hospitalization: hypertensive diseases (ICD-10: I10, I11, I15), ischemic heart disease (ICD-10: I20, I21, I22, I24, I25), congestive heart failure (ICD-10: I50), and stroke: (ICD-10: I60, I61, I62, I63, I64). In addition to these outlines, the study universe included only elective and emergency hospitalizations, in the surgical and medical specialties, and whose outcome was discharge or death. These criteria aimed at the greater homogeneity of the study population, which amounted to 385,784 hospitalizations representing 17.8% of the total number of older inpatients in the region (Figure).

**Figure:**
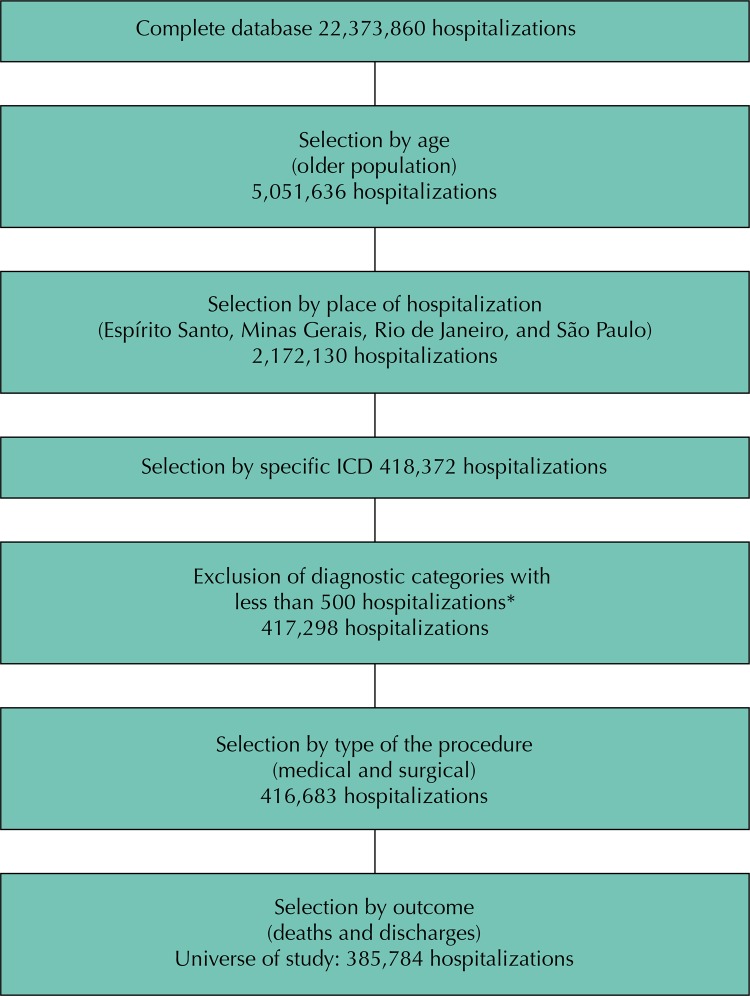
Delimitation of the study universe.

ICD: International Statistical Classification of Diseases and Related Health Conditions

* The application of the exclusion criterion of number of hospitalizations (< 500 cases) resulted in the exclusion of 1,074 observations.

The descriptive analysis considered the demographic characteristics (age and sex), the principal and secondary diagnoses, the type of admission (elective or emergency), some elements of the care process (use of the intensive care unit (ICU), medical or surgical care, and length of stay), care outcome (discharge and death), ownership (public, for-profit private, and non-profit private), and location (State) of the hospitals, as well as the place of residence of the patient and the amount reimbursed.

We applied the logistic regression to investigate the association between the occurrence of deaths and the risk factors of the patient and, later, we included the characteristics of the care process. The modeling happened in two stages: (a) construction of the model for risk adjustment of the hospital mortality considering the severity of the case (model 1) and (b) evaluation of the other variables associated with the risk-adjusted hospital death (models 2 and 3).

In the first stage, we tested the following variables: age, sex, principal diagnosis, type of admission (elective or emergency), and presence of comorbidity (secondary diagnosis). Age (six categories) and principal diagnosis (14 categories) were treated as categorical variables, and their categories of reference were, respectively, 60–64 years and congestive heart failure (ICD-10: I50). Sex and type of admission were used as dichotomous variables, and their references categories were, respectively, male and elective admission.

Given the lack of secondary diagnosis in the SIH, the presence of comorbidities was studied in three ways: (i) application of the Charlson Comorbidity Index (CCI), (ii) presence of the comorbidities proposed by Elixhauser not contemplated by the CCI, and iii) the record of another secondary diagnosis independent of the two mentioned measures. The two measures were selected for their wide use in studies with similar approach. The CCI, with 19 clinical conditions, weighs the effect of comorbidities on the prognosis of the patient^15^. The Elixhauser method, with 30 clinical conditions, was developed for use in administrative databases seeking completeness in the measurement of comorbidity for risk adjustment^16^. There is overlap between the clinical conditions included in these two measures of case severity^17^. The calculation of the CCI score and the presence of Elixhauser comorbidities were based on the algorithm developed by Quan et al.^17^, which codifies each clinical condition according to ICD-10 codes. The CCI was grouped according to frequency, as follows: score equal to zero (reference category), which means absence of severity, score equal to one, and a score equal to or greater than two. Each of the Elixhauser comorbidities were treated separately as dichotomous variables (absence = 0; presence = 1); the comorbidities that were statistically significant remained in the final model (p < 0.05): anemia, arrhythmia, hypertension, hyperthyroidism, weight loss, and other neurological diseases (model 1). Although excluded from the two measures of comorbidity, pneumonia (ICD-10: J18) was included in model 1 because of its frequency and importance in the study population. The record of the other secondary diagnoses was treated as a dichotomous variable.

In the second stage of the modeling, considering the frequency distribution, we included the length of stay, treated as a categorical variable with six ranges: up to one day (category of reference), 2–7 days, 8–15 days, 16–30 days, 31–45 days, 45–309 days (model 2). Subsequently, we included the other variables of the care process: surgery (yes; no) and use of intensive care unit (yes; no) (model 3). We evaluated the predictive ability of the models based on C-statistic, according to which values between 0.70–0.80 represent a reasonable discriminating power between predicted and observed deaths^18^.

We analyzed the risk-adjusted mortality rate (number of predicted deaths divided by hospitalizations per 100) aggregately at the hospital level, using the predicted deaths in models 1 and 2 of the logistic regression. We described the crude hospital mortality rate (TMHB) and the risk-adjusted hospital mortality rate (TMHA) as to their variation in relation to the hospital ownership and State. Based on the predicted deaths in model 1 of the logistic regression, we also calculated the hospital standardized mortality ratio (ratio between observed and predicted deaths per 100), in which values above 100 indicate worse performance and values below 100 indicate better performance. At this stage, the analysis was based on the comparison between hospitals that presented a mortality ratio higher than zero.

To analyze the data, we used the Statistical Package for the Social Science (SPSS), version 17.0.

Although we use a public and free information source, this study was submitted and approved by the Research Ethics Committee of the Escola Nacional de Saúde Pública (Process April 4, 2015).

## RESULTS

Of the 385,784 hospitalizations analyzed, 87% ended in discharges and 13% in deaths (Table 1). Mean age was 72.9 years and 51.1% of the hospitalized persons were men. The TMHB was slightly higher in females (13.6%) and more expressive in the age groups of 85–90 years (20.7%) and 91 years or more (25.4%) (Table 1). Heart failure was responsible for most of the hospitalizations among the selected diagnoses (36.8%); however, the highest TMHB was among the hemorrhagic cerebrovascular diseases (Table 1).

**Table 1:** Characteristics of the older inpatients and crude hospital mortality rate. Specific circulatory diseases, Southeast region, Brazil, 2011–2012.

Characteristics	Hospitalizations (%)	Number of deaths (crude mortality rate %)
Number of cases (%)	385,784 (100.0)	50,317 (13.0)
Sex		
Male (%)	196,975 (51.1)	24,702 (12.5)
Female (%)	188,809 (48.9)	25,615 (13.6)
Mean age (years and standard deviation)	72.91 (8.6)	75.6 (9.1)
Age group in years (n, %)		
60–64	78,378 (20.3)	6,866 (8.8)
65–70	90,219 (23.4)	9,328 (10.3)
71–74	59,296 (15.4)	7,187 (12.1)
75–80	77,239 (20.0)	11,088 (14.4)
81–84	39,142 (10.1)	6,770 (17.3)
85–90	31,036 (8.0)	6,413 (20.7)
91–100	10,474 (2.7)	2,665 (25.4)
Principal diagnosis (%)		
Hypertensive diseases		
I10 Essential hypertension (primary)	31,901 (8.3)	717 (2.2)
I11 Hypertensive heart disease	2,266 (0.6)	94 (4.1)
I15 Secondary hypertension	4,260 (1.1)	167 (3.9)
Ischemic heart disease		
I20 Angina pectoris	50,584 (13.1)	1,579 (3.1)
I21 Acute myocardial infarction	38,247 (9.9)	7,844 (20.5)
I22 Recurrent acute myocardial infarction	944 (0.2)	164 (17.4)
I24 Other acute ischemic heart diseases	16,214 (4.2)	660 (4.1)
I25 Chronic ischemic heart disease	8,536 (2.2)	454 (5.3)
Congestive heart failure (%)		
I50 Congestive heart failure	141,831 (36.8)	18,739 (13.2)
Stroke		
I60 Subarachnoid hemorrhage	1,867 (0.5)	621 (33.3)
I61 Intracranial hemorrhage	5,770 (1.5)	1,951 (33.8)
I62 Other nontraumatic intracranial hemorrhages	1,572 (0.4)	587 (37.3)
I63 Cerebral infarction	6,980 (1.8)	1,453 (20.8)
I64 Stroke not specified as hemorrhagic or ischemic	74,812 (19.4)	15,287 (20.4)
Residents of the Southeast region (%)		
Yes	385,328 (99.9)	50,266 (13.0)
Type of admission		
Elective	37,428 (9.7)	2,432 (6.5)
Emergency	348,356 (90.3)	47,885 (13.7)
Comorbidities		
Yes	53,177 (13.8)	9,915 (18.6)
Charlson Comorbidity Index (score)		
Index = 0	372,765 (96.6)	47,920 (12.9)
Index = 1	10,487 (2.7)	1,703 (16.2)
Index ≥ 2	2,532 (0.7)	694 (27.4)
Elixhauser Comorbidity		
Absence	354,933 (92.0)	45,443 (12.8)
Presence	30,851 (8.0)	4,874 (15.8)
Care Process		
Type of procedure (%)		
Medical	334,225 (86.6)	46,925 (14.0)
Surgical	51,559 (13.4)	3,392 (6.6)
Use of the ICU (%)		
Yes	69,798 (18.1)	15,656 (22.4)
No	315,986 (81.9)	34,661 (11)
Length of stay		
Mean (SD) – days	6.88 (8.3)	8.93 (12.3)
Range (days)		
≤ 1	40,380 (10.5)	10,808 (26.8)
2–7	240,074 (62.2)	21,246 (8.8)
8–15	70,588 (18.3)	10,122 (14.3)
16–30	26,363 (6.8)	5,581 (13.0)
31–45	5,544 (1.4)	1,503 (27.1)
45–309	2,835 (0.7)	1,057 (37.3)
Total reimbursement value (R$)		
Mean (SD)	2,020.75 (3,415.80)	2,548.60 (4,486.16)
Mode	699.46	669.46
Median	731.46	848.19
Variation	21.98–73,385.46	40.38–73,385.46
Hospital Ownership		
Public	143,885 (37,3)	24,562 (17.1)
For-profit Private	26,271 (6,8)	1,662 (6.3)
Non-profit Private	215,628 (55.9)	24,093 (11.2)
State		
Minas Gerais (%)	117,604 (30.5)	13,065 (11.1)
Espírito Santo (%)	16,121 (4.2)	1,749 (10.8)
Rio de Janeiro (%)	55,352 (14.3)	9,271 (16.7)
São Paulo (%)	196,707 (51.0)	26,232 (13.3)

SD: standard deviation; ICU: intensive care unit

Source: Ministry of Health - Hospital Information System of the SUS (SIH/SUS).

Most of the admissions were an emergency (90.3%), and their TMHB (13.7%) was higher than for elective admissions (6.5%). Most hospitalizations (96.6%) presented a less severe profile according to the CCI score and 92% did not register any of the Elixhauser comorbidities. However, TMHB was higher in both when the conditions were present (Table 1). Despite this, the record of secondary diagnosis (13.8%) was low.

There was a predominance of older adults hospitalized for medical care (86.6%) with TMHB (14.0%) higher than for surgical care (6.6%). Approximately 60% of the hospitalized persons stayed in the hospital between two and seven days. The mean length of stay was 6.9 days, but it was higher in deaths; comparatively, TMHB was higher in hospitalizations for more than 45 days (37.3%). The use of the ICU was low (1.2%), but the TMHB was higher in the cases that used the ICU (22.4%). Mean reimbursement value was higher in deaths (R$2,548.60) than in all hospitalizations (R$2,020.75).

Most hospitalizations occurred in non-profit private hospitals (55.9%) and public hospitals (37.3%); however, TMHB was higher in public hospitals (17.1%) (Table 1). Hospitalizations occurred mainly in the states of São Paulo (51.0%) and Minas Gerais (30.5%), but Rio de Janeiro had the highest TMHB (16.7%) (Table 1).

In the modeling to predict the deaths, the risk model (model 1) presented good discrimination (C = 0.720) and all variables related to patient risk were significant (Table 2). This model showed a higher risk of death proportional to increasing age, CCI score, and emergency admissions (Table 2). The adjusted risk of death was higher in hospitalizations in which the principal diagnosis was other nontraumatic intracranial hemorrhages (OR = 4.198), subarachnoid hemorrhage (OR = 4.034), and intracranial hemorrhage (OR = 3.799). The adjusted odds ratio for death was also higher in hospitalizations with comorbidities (OR = 2.136), low weight (OR = 1.82), and pneumonia (OR = 1.494) (Table 2).

**Table 2:** Logistic regression model: predictive factors and factors associated with in-hospital death for specific diseases of the circulatory system in older adults. Southeast region, Brazil, 2011–2012.

Variable	Model 1^c^		Model 2^d^		Model 3^e^	
		
	OR	95%CI	OR	95%CI	OR	95%CI
Sex						
Male	1	-	1	-	1	-
Female	1.041	1.021–1.062	1.043	1.022–1.064	1.042	1.021–1.064
Age group (years)						
60–64	1	-	1	-	1	-
65–70	1.179	1.140–1.219	1.184	1.144–1.226	1.190	1.148–1.233
71–74	1.382	1.333–1.433	1.409	1.358–1.463	1.434	1.381–1.490
75–80	1.637	1.584–1.692	1.661	1.605–1.719	1.697	1.639–1.757
81–84	1.959	1.886–2.034	1.991	1.916–2.070	2.072	1.992–2.156
85–90	2.378	2.287–2.472	2.428	2.333–2.527	2.556	2.453–2.663
91–100	3.089	2.930–3.256	3.219	3.048–3.400	3.501	3.311–3.703
Principal diagnosis						
I50 Congestive heart failure	1	-	1	-	1	-
Hypertensive diseases						
I10 Essential hypertension (primary)	0.155	0.143–0.167	0.086	0.080–0.093	0.087	0.080–0.094
I11 Hypertensive heart disease	0.323	0.262–0.397	0.201	0.162–0.249	0.190	0.153–0.236
I15 Secondary hypertension	0.306	0.261–0.357	0.203	0.173–0.238	0.207	0.177–0.244
Ischemic heart disease						
I20 Angina pectoris	0.264	0.250–0.278	0.196	0.186–0.207	0.203	0.192–0.215
I21 Acute myocardial infarction	1.938	1.881–1.998	1.548	1.500–1.598	1.128	1.090–1.168
I22 Recurrent acute myocardial infarction	1.733	1.461–2.056	1.470	1.230–1.757	1.073	0.890–1.293
I24 Other acute ischemic heart diseases	0.390	0.359–0.423	0.305	0.280–0.332	0.310	0.284–0.339
I25 Chronic ischemic heart disease	0.458	0.416–0.505	0.283	0.256–0.313	0.357	0.321–0.398
Stroke						
I60 Subarachnoid hemorrhage	4.034	3.652–4.456	3.272	2.950–3.630	3.205	2.878–3.570
I61 Intracranial hemorrhage	3.799	3.586–4.025	3.294	3.102–3.498	3.473	3.264–3.697
I62 Other nontraumatic intracranial hemorrhages	4.198	3.778–4.665	3.691	3.308–4.118	4.898	4.356–5.508
I63 Cerebral infarction	1.784	1.678–1.896	1.628	1.528–1.734	1.773	1.662–1.891
I64 Stroke not specified as hemorrhagic or ischemic	1.793	1.750–1.836	1.685	1.644–1.727	1.740	1.697–1.785
Type of admission						
Elective	1	-	1	-	1	-
Emergency	1.297	1.238–1.359	1.601	1.527–1.680	1.424	1.355–1.496
Comorbidity index						
Score = 0	1	-	1	-	1	-
Score = 1	1.655	1.565–1.750	1.514	1.430–1.604	1.515	1.428–1.606
Score ≥ 2	2.913	2.656–3.195	2.571	2.337–2.828	2.463	2.232–2.717
Presence of comorbidity						
No	1	-	1	-	1	-
Yes	2.136	2.054–2.222	1.876	1.800–1.954	1.965	1.884–2.049
Elixhauser Comorbidity^a^ (no/yes)						
Arrhythmia	0.772	0.679–0.878	0.771	0.675–0.881	0.681	0.594–0.781
Hypertension	0.419	0.395–0.446	0.450	0.422–0.479	0.460	0.431–0.490
Other neurological disease	0.505	0.371–0.686	0.499	0.363–0.685	0.489	0.354–0.677
Hypothyroidism	0.254	0.116–0.554	0.248	0.112–0.552	0.221	0.098–0.496
Weight loss	1.822	1.209–2.745	1.756	1.142–2.700	2.021	1.313–3.113
Deficiency anemia	0.298	0.136–0.652	0.302	0.136–0.667	0.369	0.167–0.816
Another specific prevalent comorbidity: Pneumonia^b^ (no/yes)	1.494	1.377–1.620	1.481	1.361–1.610	1.467	1.346–1.599
Length of stay (days)						
≤ 1			1	-	1	-
2–7			0.150	0.145–0.154	0.126	0.122–0.130
8–15			0.226	0.218–0.234	0.161	0.155–0.167
16–30			0.348	0.334–0.363	0.222	0.213–0.232
31–45			0.480	0.448–0.514	0.288	0.268–0.310
45–309			0.742	0.681–0.810	0.437	0.399–0.479
Use of ICU						
No					1	-
Yes					4.095	3.986–4.207
Type of care						
Medical					1	-
Surgical					0.390	0.371–0.410
Constant	0.07		0.263		0.292	

Source: Ministry of Health – Hospital Information System of the SUS (SIH/SUS).

OR: Odds ratio; 95%CI: 95% confidence interval; ICU: intensive care unit

^a^ Dichotomous variable, absence/presence of Elixhauser comorbidity.

^b^ Dichotomous variable, absence/presence of pneumonia.

^c^ Predictive capacity of model 1: C-statistic = 0.720; 95%CI 0.717–0.722.

^d^ Predictive capacity of model 2: C-statistic = 0.768; 95%CI 0.766–0.771.

^e^ Predictive capacity of model 3: C-statistic = 0.795; 95%CI 0.793–0.797.

In model 2, we observed a protective effect for hospitalizations lasting more than one day, but this effect gradually decreased as the number of hospitalized days increased. The other variables remained significant when compared to model 1 (Table 2) and there was no significant difference in the odds ratio for death between the two models. This modeling presented a better predictive ability than the previous model (C = model 2 = 0.768) (Table 2).

In the final stage of the modeling (model 3), only the odds ratio of recurrent myocardial infarction lost significance after including surgery and use of ICU (95%CI 0.890–1.293). The process variables analyzed indicated a higher chance of death in hospitalizations that used ICU (OR = 4.095) and a protective effect for surgical care (OR = 0.390), which seems to reflect a higher severity of the cases (Table 2).

Of the 1,247 hospitals analyzed, 1,115 of them had a hospital standardized mortality ratio (HSMR) other than zero. In these 1,115 hospitals, HSMR was higher in for-profit private institutions (118.2) (Table 3). In public hospitals, TMHB decreased after adjustment, even when adjusted for the days of stay (Table 3). In all hospital types, there was a significant variation in the risk-adjusted hospital mortality rate (TMHA): between 2.7% and 34.2% for public hospitals, between 2.3% and 19.2% for for-profit private hospitals, and between 1.3% and 31.8% for non-profit private hospitals. The introduction of length of stay in the adjustment of hospital mortality decreased the rates in all types of hospital. These hospitals also presented heterogeneity regarding the volume of hospitalizations; however, hospital mortality was not stratified by the volume category (Table 3).

**Table 3:** Variation of the crude and risk-adjusted hospital mortality rate according to the hospital ownership, in older inpatients for specific diseases of the circulatory system. Southeast region, Brazil, 2011–2012.

Ownership	Characteristics of the hospitals^a^	
Public	Number of hospitals	357
	Mean volume of cases (SD)	399.2 (533.8)
	Volume variation	1–4,959
	Crude hospital mortality rate (variation)	16.7 (0.80–100.00)
	Risk-adjusted hospital mortality rate 1 (variation)^b^	14.4 (2.70–34.18)
	Adjusted hospital mortality rate 2 (variation)^c^	12.7 (1.27–31.85)
	Hospital standardized mortality ratio (95%CI)	116.4 (108.32–124.20)
	Ratio variation	5.9–824.4
	Median	104.9
	SD	76.2
For-profit Private	Number of hospitals	73
	Mean volume of cases (SD)	361.4 (483.34)
	Volume variation	2–2,333
	Crude hospital mortality rate (variation)	10.0 (0.40–100.00)
	Risk-adjusted hospital mortality rate 1 (variation)^b^	11.1 (2.26–19.24)
	Adjusted hospital mortality rate 2 (variation)^c^	10.1 (30.04–28.60)
	Hospital standardized mortality ratio (95%CI)	118.2 (39.20–197.12)
	Ratio variationo	4.5–2,892.6
	Median	59.3
	SD	338.4
Non-profit Private	Number of hospitals	685
	Mean volume of cases (SD)	311.95 (475.45)
	Volume variation	1–7,521
	Crude hospital mortality rate (variation)	11.6 (0.47–100.00)
	Risk-adjusted hospital mortality rate 1 (variation)^b^	12.3 (1.27–31.84)
	Adjusted hospital mortality rate 2 (variation)^c^	11.9 (0.96–41.51)
	Hospital standardized mortality ratio (95%CI)	103.3 (80.42–126.12)
	Ratio variation	3.8–7,885.2
	Median	85.7
	SD	304.6

Source: Ministry of Health – Hospital Information System of the SUS (SIH/SUS).

95%CI: 95% confidence interval; SD: standard deviation

^a^ Including only hospitals with a mortality ratio > 0, n = 1,115 hospitals.

^b^ Deaths predicted by risk model 1 (Table 2, model 1).

^c^ Deaths predicted by risk model 2 (Table 2, model 2).

Regarding the variation in hospital mortality among the states of the Southeast region, the states of Rio de Janeiro and São Paulo had the highest TMHB, as well as higher variation in these rates (Table 4). After risk adjustment, the variation in TMHB between 10.3% (Espírito Santo) and 17.7% (Rio de Janeiro) decreased; TMHA ranged from 12.1% to 13.1% (Table 4). The risk adjustment approximated the States’ values, reversing the previous condition that showed Rio de Janeiro and São Paulo with the highest TMHB, again emphasizing the importance of the adjustment (Table 4).

**Table 4:** Variation of the crude and adjusted hospital mortality rate by State, in older adults hospitalized for specific diseases of the circulatory system. Southeast region, Brazil, 2011–2012.

State	Number of hospitals^a^ (%)	Crude hospital mortality rate (variation)	Risk-adjusted hospital mortality rate 1^b^ (variation)	Adjusted hospital mortality rate 2^c^ (variation)
Minas Gerais	441 (39.6)	10.9 (0.53–100.00)	13.1 (2.82–26.64)	12.0 (2.87–41.51)
Espírito Santo	60 (5.4)	10.3 (0.58–40.00)	12.1 (5.28–32.60)	11.5 (4.33–33.44)
Rio de Janeiro	165 (14.8)	17.7 (0.40–100.00)	13.1 (1.27–34.18)	13.5 (0.96–35.23)
São Paulo	449 (40.3)	14.0 (0.47–100.00)	13.1 (2.37–27.22)	13.0 (1.40–28.47)

Source: Ministry of Health – Hospital Information System of the SUS (SIH/SUS).

^a^ Including only hospitals with a mortality ratio > 0, n = 1,115 hospitals.

^b^ Deaths predicted by risk model 1 (Table 2, model 1).

^c^ Deaths predicted by risk model 2 (Table 2, model 2).

## DISCUSSION

In addition to the analysis of factors associated with the outcome of hospital care provided to older inpatients in the Brazilian Southeast region in the SUS, we explored TMHA as an indicator of the effectiveness of care^13^. Despite the limits, given the lack of information, the risk adjustment model presented a reasonable ability for discrimination. In addition, the analysis indicated that the length of stay predicted the risk of death. There was a clear improvement comparing the predictive ability of the models with the inclusion of the length of stay: C-statistic increased from 0.72 in model 1 to 0.77 in model 2. Although this finding is different from that found in a Japanese’ study, whose inclusion of length of stay decreased the discriminative power of the model^19^, the results illustrate the complexity and difficulty of interpreting the relationship between death and hospital stay. A protective effect was observed for hospitalizations lasting more than one day, possibly related to the severity of the case at the moment of admission or the inadequacy of the emergency care, which strictly requires timely and appropriate actions. This effect gradually decreased as the days of hospitalization increased; similar results have been observed in older adults hospitalized in Japan^19^ and among beneficiaries of the Medicare program in the United States^20^.

Women had a slightly higher chance of dying (OR = 1.04). This result differs from Amaral et al.^11^ and the methodological differences between both studies could be one of the reasons. These authors have found no significant association in TMHA for sex in older adults hospitalized in four hospitals in Rio de Janeiro. The increase in the chance of death according to age was in line with the expectations and what has been reported in several studies^11^ ^,^ ^19^.

Compared to the other selected reasons, stroke presented the highest TMHB and greater odds ratio, which is consistent with the severity of the diseases and described in Brazilian studies^12,21–23^. Regarding the comorbidity indexes, the greater chance of death when the CCI score was equal to or greater than two indicated greater severity, similar to previous studies^15^ ^,^ ^19^. Of the Elixhauser comorbidities, the presence of low weight (OR = 1.82) can be highlighted, as the other comorbidities had a protective effect. However, the quality of the information impacts the accuracy of this type of index. In addition, pneumonia (OR = 1.49) presented a higher risk of death, but we were not able to determine if it was present at admission or if it occurred during hospitalization, i.e., we could not say if it was a comorbidity or an avoidable complication.

Furthermore, as expected, we observed a higher risk of death in older adults who used the ICU (OR = 4.095), as described in another study^21^. This finding may express the greater case severity forwarded to the ICU. However, aspects related to access to these beds and the care process influence the use of this resource^21^. In general, studies have reported a significant association and protective effect between admissions directly forwarded to the ICU and greater chances of survival in the adult population^24^ ^,^ ^25^.

At the hospital level, TMHB was higher in public hospitals (16.7%) than in non-profit private (11.6%) and for-profit private hospitals (10.0%). The adjustment of this indicator decreased TMHA only in public hospitals, highlighting the importance of risk adjustment. There is great variability in TMHA among hospitals, which raises the hypothesis of possible problems related to the quality of hospital care. As for HSMR, it was slightly higher in for-profit private hospitals (118.2%) than in public hospitals (116.4%), but both performed worse than expected (> 100). These results show the need for detailed analysis of this variation and continuous performance monitoring.

However, we need to consider the limits of this study, especially regarding the robustness of hospital mortality as an indicator of the quality of care, mainly related to the causal validity between process and outcome and the precision of the risk adjustment^6^ ^,^ ^7^. Separating the variation because of case severity from the care process and the clinical performance of the professionals and the organization is an even more complex task in older inpatients, in which these elements may be more imbricated^5–7^. However, this type of approach is understood as a screening instrument, that is, an alert signal that requires subsequent analyses in order to improve the effectiveness of care and, consequently, its quality^6^ ^,^ ^12^.

Another important limitation refers to the structure of the SIH, which contained only one field for secondary diagnosis’ record in the studied period, besides the underreporting, coverage, and quality of the data available. In this study, we observed low filling of the secondary diagnosis, namely, 13.8%. This value was lower than that described by Amaral et al.^11^, who have found a record in 19.5% of the hospitalizations in the state of Rio de Janeiro; however, our value was higher than that found by Martins^15^ (5.4%) in the hospitalizations throughout Brazil. These shortcomings, especially related to the description of comorbidities and complications, impact the analyses performed.

In Brazil, the evaluation of hospital care using administrative data and risk-adjusted performance indicators is a poorly developed subject^13^. There is also a shortage of studies, specifically, on the hospital care provided to older adults. Thus, new methodologies and analyses need to be elaborated on the effects of the multiplicity of chronic diseases, which affect older adults more intensely^6^. Considering this context, this work contributes to the Brazilian production, as it analyzed the quality of hospital care for older patients using SIH data and explored the CCI and, complementarily, the Elixhauser comorbidities for risk-adjusted hospital mortality. Although the source of information presented problems during the study period, the use of these indexes along with the other variables could acceptably predict the hospital death of older adults, and it could be improved in the future to monitor the quality of care provided.

On the other hand, despite the contribution made, the development of new research is essential to increase the knowledge on the profile of hospital interventions performed in older patients in Brazil and their effectiveness. There is a lack of analyses on care integration aimed at this age group, considering the prevalence of multi-comorbidities, the effectiveness of primary care, and the access to specialized services, whose actions are fundamental to prevent the worsening of cases and even hospitalization. Therefore, further studies and monitoring initiatives would help identify problems in the process of the hospital care, as well as subsidize and encourage good practices and programs that can make the health care model more suited to the needs and demands of the older adult throughout the care line – before and after hospitalization.

## Footnotes

a Ministério da Saúde (BR). DATASUS: morbidade hospitalar do SUS (SIH/SUS). Brasília (DF); 2015 [cited 2015 Jun 26]. Available from: http://www2.datasus.gov.br/DATASUS/index.php?area=0203&id=6926
